# A Thioredoxin Domain-Containing Protein Interacts with *Pepino mosaic virus* Triple Gene Block Protein 1

**DOI:** 10.3390/ijms19123747

**Published:** 2018-11-25

**Authors:** Matthaios M. Mathioudakis, Souheyla Khechmar, Carolyn A. Owen, Vicente Medina, Karima Ben Mansour, Weronika Tomaszewska, Theodore Spanos, Panagiotis F. Sarris, Ioannis C. Livieratos

**Affiliations:** 1 Mediterranean Agronomic Institute of Chania, Department of Sustainable Agriculture, Alsylio Agrokepio, GR-73100 Chania, Greece; manth82@yahoo.gr (M.M.M.); kh.souhila@live.fr (S.K.); owen@maich.gr (C.A.O.); karina79@hotmail.fr (K.B.M.); ikatomaszewska@gmail.com (W.T.); theodor@maich.gr (T.S.); 2 Departament de Producció Vegetal i Ciència Forestal, Universitat de Lleida, 25198 Lleida, Spain; medinap@pvcf.udl.cat; 3 Institute of Molecular Biology and Biotechnology, Foundation for Research and Technology-Hellas, GR-70013 Heraklion, Greece; p.sarris@imbb.forth.gr; 4 Department of Biosciences, College of Life and Environmental Sciences, University of Exeter, Exeter EX4 4QD, UK

**Keywords:** potexviruses, *Pepino mosaic virus*, host-pathogen interactions, thioredoxins, phosducin-like proteins

## Abstract

*Pepino mosaic virus* (PepMV) is a mechanically-transmitted tomato pathogen of importance worldwide. Interactions between the PepMV coat protein and triple gene block protein (TGBp1) with the host heat shock cognate protein 70 and catalase 1 (CAT1), respectively, have been previously reported by our lab. In this study, a novel tomato interactor (*Sl*TXND9) was shown to bind the PepMV TGBp1 in yeast-two-hybrid screening, in vitro pull-down and bimolecular fluorescent complementation (BiFC) assays. *Sl*TXND9 possesses part of the conserved thioredoxin (TRX) active site sequence (W__PC vs. WCXPC), and TXND9 orthologues cluster within the TRX phylogenetic superfamily closest to phosducin-like protein-3. In PepMV-infected and healthy *Nicotiana benthamiana* plants, *Nb*TXND9 mRNA levels were comparable, and expression levels remained stable in both local and systemic leaves for 10 days post inoculation (dpi), as was also the case for catalase 1 (CAT1). To localize the TXND9 in plant cells, a polyclonal antiserum was produced. Purified α-*Sl*TXND9 immunoglobulin (IgG) consistently detected a set of three protein bands in the range of 27–35 kDa, in the 1000 and 30,000 *g* pellets, and the soluble fraction of extracts of healthy and PepMV-infected *N. benthamiana* leaves, but not in the cell wall. These bands likely consist of the homologous protein *Nb*TXND9 and its post-translationally modified derivatives. On electron microscopy, immuno-gold labelling of ultrathin sections of PepMV-infected *N. benthamiana* leaves using α-*Sl*TXND9 IgG revealed particle accumulation close to plasmodesmata, suggesting a role in virus movement. Taken together, this study highlights a novel tomato-PepMV protein interaction and provides data on its localization in planta. Currently, studies focusing on the biological function of this interaction during PepMV infection are in progress.

## 1. Introduction

*Pepino mosaic virus* (PepMV; genus *Potexvirus*) is a mechanically-transmitted virus, which has evolved in only a few years from an emerging to important endemic pathogen in several tomato (*Solanum lycopersicum)*-growing areas of the world [1]. PepMV-induced symptomatology in tomato varies from mild or severe yellowing to necrosis, dependent on the specific virus strain, and accordingly, the economic impact of PepMV epidemics can vary considerably [2,3]. The PepMV genome consists of a single-stranded, positive sense RNA (6400 nt) that includes two short flanking untranslated regions (UTRs) with a methylguanosine cap structure and a poly(A) tail at the 5’- and 3’-ends, respectively [4]. The 3’-UTR can fold into three independent stem-loops [5]. In common with all potexviruses, five open reading frames (ORFs) encode the replicase protein (164 kDa; displaying methytransferase, helicase/NTP-binding and RNA-dependent RNA polymerase motifs (RdRp)), three ‘triple gene block’ (TGB) proteins of 26 (TGBp1; p26), 14 (TGBp2; p14) and 9 (TGBp3; p9) kDa, and the coat protein (CP; 25 kDa) [4]. The viral replicase is expressed from the genomic RNA, while TGBp1-3 and the CP are expressed via 3’ co-terminal subgenomic RNAs [6]. The importance of PepMV-induced diseases in commercial tomato cultivation has resulted in the production of specific molecular tools such as infectious cDNA clones to facilitate studies on virus biology, pathogenesis and reverse genetics [6,7].

One goal of plant virology research is to identify viral and host components involved in infection, and elucidate the molecular mechanisms underlying defense, virulence and susceptibility. Various host-virus interactions have previously been reported for the prototype *Potato virus* X (PVX) and other potexviruses, where the TGBp1 homologues interact with host proteins that affect symptom response, virus multiplication and accumulation, viral movement, phloem unloading and protein phosphorylation [8,9]. For example, PVX TGBp2 increases the plasmodesmal size exclusion limit to facilitate virus movement following interaction with the TIP host factor, and callose degradation in infected cells [10]. For PepMV, a study by Hanssen et al. [11] showed that tomato seedlings infected by two different PepMV strains (mild and severe) differentially up- and down-regulate the transcription of different host gene groups suggesting the existence of distinct PepMV strain-dependent interactions with their host. For example, an RdRp domain represents a necrosis-inducing elicitor [12] whereas specific amino acids located in TGBp3 and CP act as symptom determinants [3,13]. Part of or the entire PepMV CP acts as a pathogenicity determinant or an elicitor of the *Rx* gene based resistance [14,15]. PepMV CP binds the heat-shock cognate protein 70 (Hsc70) isoform 3 to facilitate PepMV infection [16,17], whereas TGBp1 interacts with tomato catalase 1 (CAT1) to elevate its hydrogen peroxide scavenging efficiency, most likely to regulate plant defense mechanisms and facilitate virus accumulation [18]. Recently, *Bamboo mosaic virus* (BaMV) TGBp2 was shown to interact with a *Nicotiana benthamiana*-expressed thioredoxin type h protein (*Nb*TRXh2), which acts as a negative regulator of virus movement [19].

The TRX superfamily is a large group of redox proteins displaying similar secondary structures that share one or several common structural motifs—“thioredoxin domains”—that encompasses five major subfamily groups: TRXs, glutaredoxins (GRXs), protein disulphide isomerases (PDIs), glutathione peroxidases (GPs) and glutathione S-transferases (GSTs), in addition to their reductants [20,21]. TRXs constitute a diverse group of small proteins found in all organisms that regulate the redox status of target proteins using a conserved amino acid motif (WCXPC) that catalyzes the dithiol-disulphide exchange reaction [21]. In *Arabidopsis thaliana*, seven TRX types are located in various cellular compartments such as chloroplasts, mitochondria, plastids and the cytosol [21,22,23]. TRXs have been shown to participate in (and affect) multiple processes including gene expression, signal transduction, proliferation and apoptosis, and in particular tolerance of oxidative stress. In the latter, TRXs may play an active role as scavengers of reactive oxygen species (ROS) or participate in signaling within the antioxidant network [24].

Here, we report the identification of a TRX-like tomato protein (*Sl*TXND9) that interacts with PepMV TGBp1. Like CAT1, *Nb*TXND9 expression is not appreciably increased in PepMV-infected leaves, but the protein has been shown to accumulate in membranes and organelles close to plasmodesmata and endoplasmic reticulum of infected tissues, where potexviruses move from cell-to-cell. The manner of action of TXND9 in PepMV-infected plants remains to be addressed.

## 2. Results

### 2.1. PepMV TGBp1 Interacts with an Uncharacterized Tomato cDNA in a Yeast Library Screen Assay 

**Figure 1:**
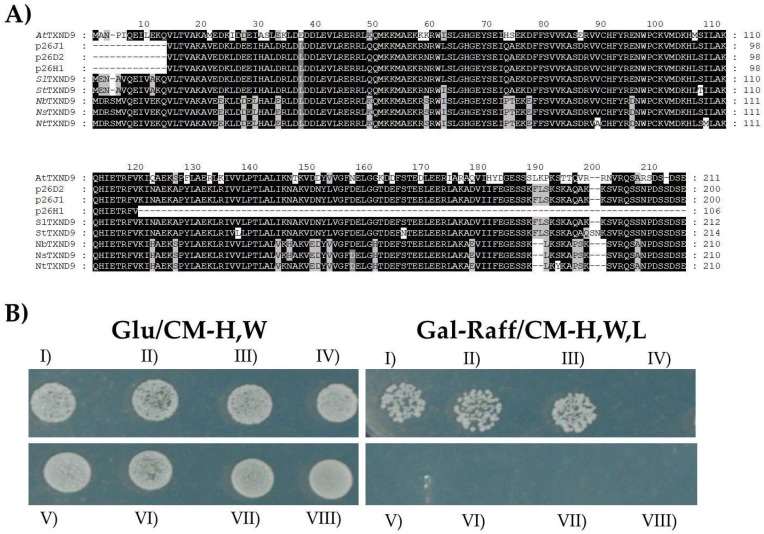
Three tomato cDNAs that interact with the *Pepino mosaic virus* (PepMV) triple gene block protein 1 (TGBp1) identified by yeast library screening. (**A**) Amino acid (aa) multiple sequence alignment of the three identified uncharacterized interactors (p26H1, p26J1, p26D2) from a tomato cDNA library and the homologous *Arabidopsis thaliana* thioredoxin domain-containing protein 9 (*At*TXND9; NP_179489.1). The full-length protein sequence obtained from *Solanum lycopersicum* (*Sl*) and the TXND9 orthologues from *Solanum tuberosum* (*St*; XP_006352322), *Nicotiana benthamiana* (*Nb*; Nbv5.1tr6261097), *Nicotiana tabacum* (*Nt*; XP_016486197) and *Nicotiana sylvestris* (*Ns*; XP_009767764) were also used. Conserved regions corresponding to the majority of the sequences are indicated with black color and with grey the different amino acids in the conserved sequences. (**B**) Interaction between PepMV TGBp1 and the isolated prey plasmids (interactors) using the yeast two-hybrid assay. Co-transformed *Saccharomyces cerevisiae* EGY48 cells grown in nonselective glucose/complete medium lacking histidine and tryptophan (Glu/CM-H,W) (**left**) and selective galactose-raffinose/complete medium lacking histidine, tryptophan and leucine (Gal-Raff/CM-H,W,L) (**right**) of the pairwise combinations: I, pGILDA-p26//pJG4-5/p26J1; II, pGILDA-p26//pJG4-5/p26H1; III, pGILDA-p26//pJG4-5/p26D2; IV, pGILDA//pJG4-5; V, pGILDA//pJG4-5/p26J1; VI, pGILDA/pJG4-5/p26H1; VII, pGILDA//pJG4-5/p26D2; VIII, pGILDA-p26//pJG4-5.

Immunoblot analysis using a specific anti-*Lex*A antibody confirmed the expression of the *LexA*-TGBp1 fusion protein in EGY48 yeast cells harboring the pJG4-5/tomato cDNA library (Figure S1A). Three positive clones (denoted p26H1, p26J1, p26D2), with the strongest LEU2^+^ phenotype, were isolated, sequenced and shown to contain inserts of 604 and 319 nucleotides. BLAST analysis revealed that the clones encoded overlapping putative polypeptides that exhibited 80% to 89% identity to a thioredoxin domain-containing protein 9 homologue of *A. thaliana* (*At*TXND9; Figure 1A). In order to verify the potential screening interaction in yeast, the three library (prey) DNA plasmids (interactors) were isolated from the yeast cells and co-transformed with EGY48 yeast cells harboring the pGILDA-p26 in a new set of two-hybrid experiments. The interactions were confirmed by the growth of co-transformed yeast cells with pGILDA-p26//pJG4-5/p26H1 or -p26J1, or -p26D2 in selective Gal-Raff/-H, W, L plates (Figure 1B). Co-transformed yeast cells with the combinations pGILDA-p26//pJG4-5, pGILDA//pJG4-5/isolated clones and pGILDA//pJG4-5 did not grow on this medium (Figure 1B).

### 2.2. Tomato SlTXND9 Clusters Phylogenetically with a Phosducin-Like Protein 3

The sequence obtained for the tomato interactor (200 amino acids) was compared (using the BLAST algorithm) with the tomato database and revealed 100% identity with a recently reported tomato cDNA clone (Acc. No, AK321611). After cloning the full-length gene sequence (Acc. No. KY798439), a preliminary alignment of *Sl*TXND9 with orthologues from *Arabidopsis thaliana*, *Solanum tuberosum*, *Nicotiana tabacum*, *Nicotiana benthamiana* and *Nicotiana sylvestris* showed a high degree of conservation between the analogous proteins of these species (75% to 96% for all *Nicotiana* species) (Figure 1A). TXND9 is also highly conserved between various unrelated plant species with protein identity levels that range from 71% (*Spinacia oleracea*-*Raphanus sativus*) to 97% (*R. sativus*-*Brassica oleracea*).

**Figure 2:**
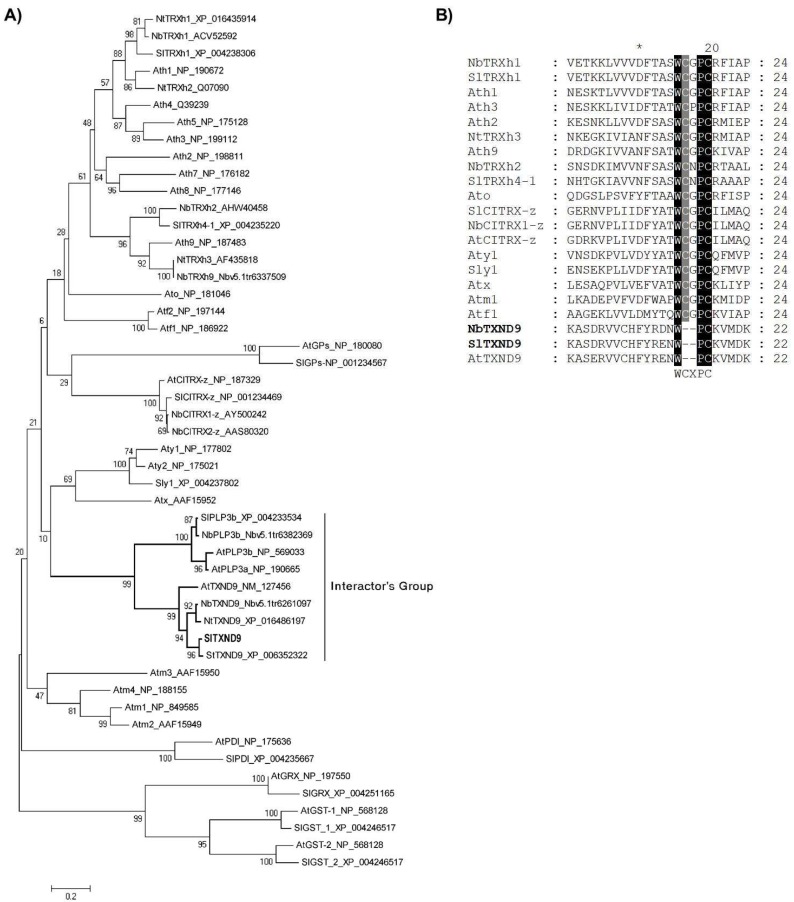
Characterization of the tomato interactor *Sl*TXND9. (**A**) A Neighbor-Joining tree generated using amino acid sequences of selected genes from the four protein groups that constitute the thioredoxin superfamily, the TXND9 orthologues and PLP3 sequences, was used to infer their evolutionary relationships. Accession numbers are given next to each gene. The analysis involved 50 amino acid sequences. The value on the left of each branch represents the percentage of replicate trees in which the associated taxa clustered together in the bootstrap test. The tree is drawn to scale, with branch lengths in the same units as those of the evolutionary distances used to infer the phylogenetic tree. (**B**) Partial amino acid sequence alignment with the tomato interactor (*Sl*TXND9) identified orthologous genes from *N. benthamiana* (*Nb*) and *A. thaliana* (*At*) and selected thioredoxin genes from *Sl*, *Nb* and *At* that possess the WCXPC active redox motif. The asterisk indicates the tenth amino acid; W--PC vs. WCXPC motifs are highlighted, with black color the conserved W, P and C amino acids in all proteins (TXND9 orthologues and thioredoxin types proteins) and with gray color the conserved C amino acid of the thioredoxin type proteins.

To examine the relationship of TXND9 with other members of the plant TRX superfamily, phylogenetic trees were generated. The full-length protein sequences of members of each of the identified types of TRX protein groups from tomato (type h, y and z) [25,26], *N. benthamiana*, *N. tabacum* and *A. thaliana* (types x, o, m, h, f, z, y) were included. The four related protein groups (GRX, PDI, GP, GST) in the TRX superfamily (Figure 2A) were also included, together with sequences from phosducin-like proteins 3 (PLP3) from *A. thaliana*, *S. lycopersicum* and *N. benthamiana*, as these shared the highest degree of amino acid identity with *Sl*TXND9 in the TAIR database, using a 1 × 10^−5^ *E*-value as a threshold.

Identical phylogenetic trees were produced whether the full-length proteins, or only the region containing the TRX domains, were compared (Figure 2A). These data clearly support the classification of the *Sl*TXND9 and its orthologues within the thioredoxin superfamily. Nevertheless, all thioredoxin types contain a conserved and active redox motif (WCXPC) [22], while for all TXND9 orthologues from the plant kingdom this motif is incomplete (W--PC) (Figure 2B), and these proteins constitute a distinct clade in the phylogenetic tree. As expected, among the proteins with a close phylogenetic relationship to TXND9 are phosducin-like protein 3 (PLP3) homologues from *A. thaliana*, and its orthologues from *S. lycopersicum* and *N. benthamiana* that share 33% to 38% amino acid identity. 

### 2.3. In Vitro Confirmation of the TGBp1-*Sl*TXND9 Interaction

**Figure 3:**
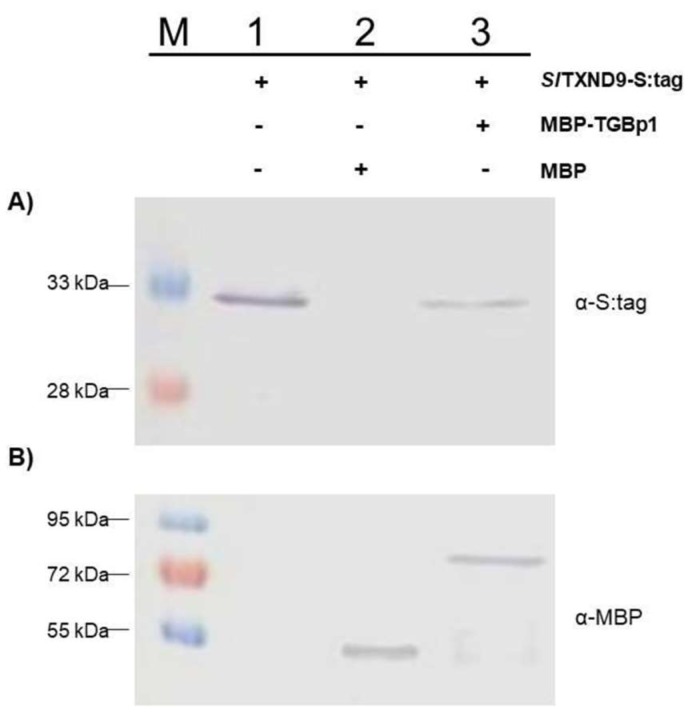
*Pepino mosaic virus* triple gene block 1 (TGBp1) interacts in vitro with *Sl*TXND9. Maltose-binding protein (MBP) and an MBP-TGBp1 fusion were expressed in *Escherichia coli* BL21 (DE3) cells along with the bacterially expressed tomato *Sl*TXND9 interactor labelled with the S:tag (*Sl*TXND9-S:tag). MBP alone (lane 2) or MBP fused with TGBp1 (MBP-TGBp1; lane 3) was amylose affinity purified and incubated with the expressed *Sl*TXND9-S:tag (lanes 1–3). The eluates were analyzed for the presence of the *Sl*TXND9, MBP or an MBP fusion using α-S:tag (**A**) or α-MBP (**B**) antibodies. The lane M indicates the protein marker, whereas the “+” and “−“ indicate the presence or absence, respectively, of the *Sl*TXND9-S:tag, MBP-TGBp1 or MBP in each lane.

To further confirm the TGBp1-*Sl*TXND9 interaction, an in vitro experiment was performed with fusion proteins incorporating either PepMV TGBp1 as (MBP-TGP1) or *Sl*TXND9 as (*Sl*TXND9-S:tag) were over-expressed in *Escherichia coli* BL21 cells. SDS-PAGE analysis of soluble cell extracts from cultures in which recombinant protein expression had been induced revealed the presence of intense protein bands of approximately 70 kDa for MBP-TGBp1 or and 30 kDa for *Sl*TXND9-S:tag, respectively. The ΜΒΡ-TGBp1 fusion and a 42.5 kDa ΜΒΡ negative control protein were affinity-purified on amylose columns before their incubation with soluble extracts containing *Sl*TXND9-S:tag. Following extensive washing, the associated proteins were eluted, Western-blotted, and subjected to analysis for the presence of MBP or S:tag moieties using specific antibodies. A 30 kDa protein band recognized by α-S:tag was detected exclusively in the eluate from the MBP-TGBp1 column (Figure 3A, lane 3) indicating the formation of an MBP-TGBp1/*Sl*TXND9-S:tag complex, while this protein was not detected in the eluate from the MBP column (Figure 3A, lane 2). These observations support the existence of a specific PepMV TGBp1/*Sl*TXND9 interaction, in agreement with the yeast assay results.

### 2.4. In Planta Localization of the SlTXND9-GFP and TGBp1:SlTXND9 Interaction

**Figure 4:**
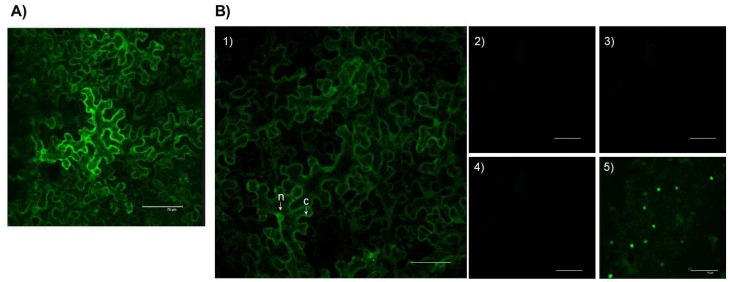
In planta subcellular localization of the *Sl*TXND9 interacting protein in *Nicotiana benthamiana* cells and bimolecular fluorescent complementation (BiFC) interaction assay. (**A**) Subcellular localization of *Sl*TXND9 fused with the green fluorescent protein (GFP). The fluorescence was visualized by confocal laser scanning microscopy in the cell cytoplasm. (**B**) Reconstitution of yellow fluorescent protein (YFP) fluorescence was visualized by confocal laser scanning microscopy after co-expression of the fusion proteins. (**B1**) Co-infiltration of pCYFP-*Sl*TXND9 and pNYFP-TGBp1 revealing their interaction in the cell cytoplasm. (**B2**–**B4**) Co-infiltrations of pCYFP with pNYFP, pCYFP-*Sl*TXND9 with pNYFP, and pCYFP with pNYFP-TGBp1 (negative controls). (**B5**) Co-infiltration of pCYFP-bZIP63 and pNYFP-bZIP63 (positive control) revealing the interaction in the nucleus. c: cytoplasm; Bars: 75 mm. The arrows in (**B1**) indicate cell wall (c) and a nucleus (n).

The *Sl*TXND9 homologue protein was over-expressed using the binary vector pROK2-GFP-HA, fused between the GFP gene and the HA tag, to facilitate examination of its subcellular tropism in planta in *Agrobacterium*-infiltrated *N. benthamiana* leaves. The expression of the GFP-*Sl*TXND9 fusion was initially confirmed by immunoblot analysis using α-GFP antibodies (Supplementary Data Figure S3), whereas the fluorescence signals derived from the protein construct were observed only in the cytoplasm of epidermal cells in agroinfiltrated leaves (Figure 4A).

The bimolecular fluorescent complementation assay (BiFC) was also used to test the TGBp1:*Sl*TXND9 interaction and to determine its subcellular localization in agroinfiltrated *N. benthamiana* plants [27]. PepMV TGBp1 and *Sl*TXND9 were fused with the N-terminal and C-terminal fragments of the Yellow Fluorescent Protein (YFP) (NYFP, CYFP), respectively. The expression of PepMV TGBp1 and *Sl*TXND9 from the constructs within infiltrated tissue was confirmed by immunoblot analysis using anti-HA and anti-*c*-myc antibodies (Figure S1B). Co-expression of pNYFP-TGBp1 and pCYFP-*Sl*TXND9 resulted in the reconstitution of functional YFP, further confirming the interaction between of TGBp1 and *Sl*TXND9 in planta. YFP fluorescence was observed throughout the cytoplasm but in no other cellular compartment (Figure 4(B1)). On co-expression of pNYFP and pCYFP, or in combination with either *Sl*TXND9 or TGBp1 fusions, no YFP fluorescence signal was detected (Figure 4(B2)–4), whereas self-interaction of bZIP63 in the nucleus (positive control; Reference [26]) resulted in YFP fluorescence (Figure 4(B5)). These results further supported a specific interaction in living epidermal cells for split YFP-tagged PepMV TGBp1 and *Sl*TXND9, occurring within the cytoplasm.

### 2.5. Measurement of NbTXND9 mRNA Levels on PepMV Infection

The possibility of regulation of *Nb*TXND9 mRNA expression in response to PepMV infection was examined. PepMV- and mock-inoculated *N. benthamiana* leaf samples (local and systemic leaves) were collected 2, 4, 6, 8, and 10 days post inoculation (dpi), and *Nb*TXND9 mRNA expression levels were evaluated using quantitative RT-PCR (qRT-PCR). No upregulation of *Nb*TXND9 mRNA expression levels was observed in PepMV-inoculated leaves (Figure S2). In previous studies, during PepMV infection, levels of the mRNA encoding an Hsc70 isoform (a PepMV CP interactor), were upregulated, whereas the mRNA levels for CAT1 (a PepMV TGBp1 interactor), were unaffected [16,18].

### 2.6. NbTXND9 Presence in Fractionated N. benthamiana Leaf Proteins

**Figure 5:**
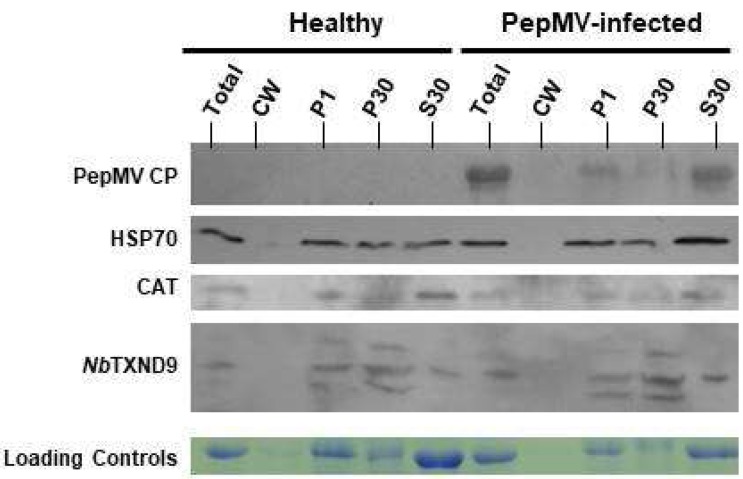
Immunoblot analysis of fractionated proteins from healthy and PepMV-infected *N. benthamiana* plants. Examination of the compartmentalization PepMV virions and the host interactors of PepMV TGP1 and coat protein (CP), CAT1 and HSP70 respectively, and *Nb*TXND9 in fractionated plant extracts. Total represents the total protein extract, CW represents the cell wall fraction; P1 is the 1000× *g* pellet fraction; P30 is the 30,000× *g* pellet fraction and S30 is the 30,000× *g* supernatant fraction. Labels on the left indicate the antiserum used to probe each panel.

Total protein extracts from healthy and PepMV-infected *N. benthamiana* leaves were fractionated before being immunoblotted to compare the presence of *Nb*TXND9, PepMV CP, and two PepMV-interacting host proteins, Hsc70 and CAT1 in each fraction (Figure 5). The subcellular fractions examined were cell wall (CW), two pellets collected at 1000× *g* (P1) and 30,000× *g* (P30); and the soluble fraction (30,000× *g*; SN). The P1 fraction is considered to mainly comprise nuclei, chloroplasts and a small proportion of the mitochondria, and P30 the majority of the mitochondria together with membranes derived from the ER. The soluble fraction is predominately cytosol, together with soluble proteins from disrupted organelles. As expected, PepMV CP was only detected in PepMV-infected plants, and was predominantly located in the soluble fraction. The α-*Sl*TXND9 IgG produced in this study reacted in more than five experiments with three proteins with sizes in the range of 27–35 kDa, which could represent post-translationally modified versions of the target *Nb*TXND9. The *Nb*TXND9, Hsc70 and CAT1 were observed in the P1 and P30 of fractions from healthy and infected leaves but the CAT1 and Hsc70 were also abundant in the soluble fraction (S30), whereas the *Nb*TXND9 was present in this fraction to a lesser degree. None of the proteins were detected in the cell wall fraction, and complete absence of detectable *Nb*TXND9 in this fraction indicates that the intense fluorescent signal seen at the cell periphery in the BiFC assay (Figure 4) represents protein accumulation in compartments immediately adjacent to the cell wall. The presence of *Nb*TXND9 in both infected and healthy leaves appeared to be identical, with no detectable difference in the amount of each protein or in its compartmentalization. The detection of equivalent amounts of *Nb*TXND9 in both healthy and PepMV-infected tissue is in agreement with the stable levels of *Nb*TXND9 transcript detected in the real-time PCR assays (Figure S2). 

### 2.7. NbTXND9 Localizes in the Plasmodesmata and ER of PepMV-Infected Leaf Cells

**Figure 6:**
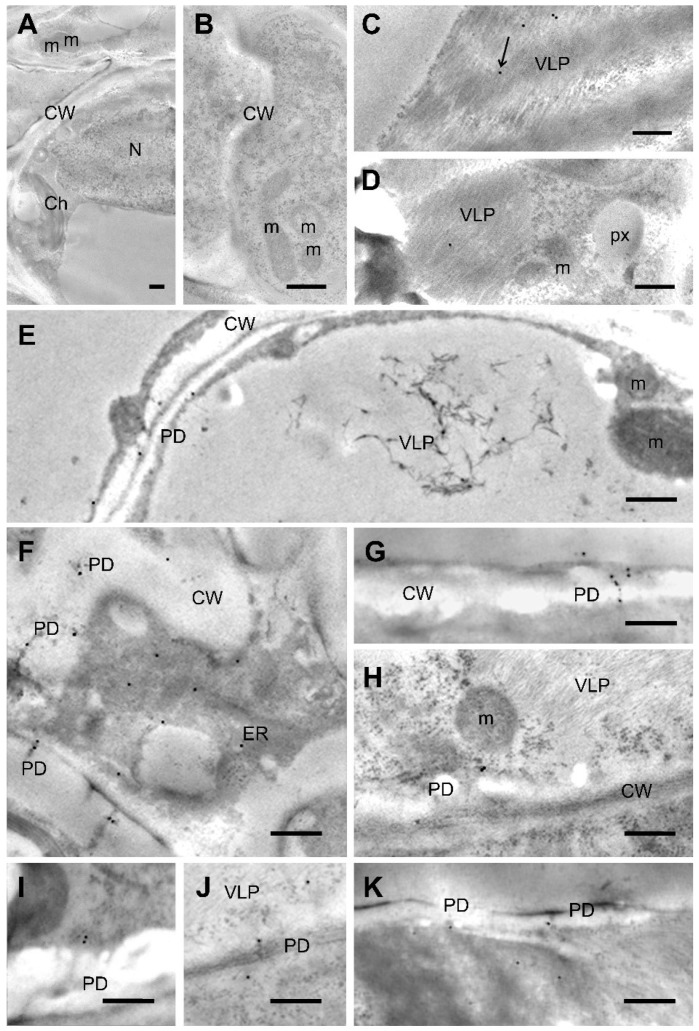
Immuno-gold labelling (IGL) of *N. benthamiana* Leaf Sections with α-*Sl*TXND9 IgG. (**A**) Healthy tissue shows no labelling. (**B**) *Cucumber mosaic virus* (CMV)-infected shows no labelling. (**C**–**K**) PepMV infected tissue. (**C**,**D**) PepMV-like particles (VLP) in systemically infected leaves showing a light labelling at 4 dpi. (**E**) Labelled scattered VLPs in the vacuole of the epidermal cell of a locally-infected leaf. Note the specific labelling of the plasmodesmata (PD). (**F**–**K**) Specific labelling of PDs (Ch: chloroplast, m: mitochondria, ER: endoplasmic reticulum, px: peroxisome, CW: cell wall, N: nucleus; arrow in C indicates a 15 nm gold particle; bars: 500 nm).

Immuno-gold labelling of ultra-thin sections of *N. benthamiana* leaves using the α-*Sl*TXND9 IgG resulted in no background in healthy tissues (Figure 6A) or in tissues infected with *Cucumber mosaic virus* (CMV) (Figure 6B), except for a very slight labelling of the chloroplasts in some cells (Figure S3). Some gold particles were observed in association with PepMV viroplasm (Figure 6C–E), and more commonly in the vicinity of and within the plasmodesmata in PepMV-infected leaves (Figure 6F–K). Taking into consideration the data from Western blot analysis, the comparable content of *Nb*TXND9 protein in PepMV-infected and healthy tissues would indicate that in the latter, the protein is widely and diffusely distributed, and that upon PepMV infection there is recruitment of *Nb*TXND9 around plasmodesmata. 

## 3. Discussion

It is known that redox status alterations in plants are associated with immune responses, and there is increasing evidence that TRXs are involved in these changes. For example, the conversion of NPR1, a master regulator of salicylic acid (SA)-mediated defense genes, from oligomers to the monomeric form has been shown to be catalyzed by TRXs, and to confer plant immunity [28]. CITRX-z is a negative regulator of the hypersensitive response (HR) in tomato and in *N. benthamiana.* When CITRX-z is silenced, ROS accumulation is enhanced, which then alters protein kinase activity and induces the expression defense-related genes, conferring resistance to *Cladosporium fulvum* [25]. In cotton, *Gb*NRX1 scavenges ROS in response to *Verticillium dahliae* infection [29], whereas cytosolic TRXs from *N. benthamiana* can activate the RipAY virulence effector in order to degrade glutathione, which affects ROS production and the expression of immunity marker genes [30]. There have also been several studies that attribute opposing roles to TRXs in pathogenesis and resistance. A chloroplastic *Nt*TRXh3 in *N. tabacum* confers resistance to *Tobacco mosaic virus* and CMV [31], whereas an atypical h type TRX from maize imparts early resistance to *Sugarcane mosaic viru*s [32] and an h type in pepper plants induces SA-related genes during *Euphorbia mosaic virus*-Yucatan Peninsula infections [33].

In this study, a previously uncharacterized tomato protein containing a TRX domain has been shown to bind PepMV TGBp1 in vitro and in vivo. The isolated tomato cDNA library clones indicated a gene orthologue of *Arabidopsis At*TXND9 with a predicted amino acid sequence identity of 75%. Further analysis revealed that *Sl*TXND9 clusters within the TRX superfamily and shares a high degree of conservation to orthologues of other plants that all possess only a part (W--PC) of the characteristic TRX redox motif (WCXPC). Interestingly, our phylogenetic analysis also revealed that the TXND9 homologues cluster most closely with PLP3, in a distinct branch which is divided into two closely related sub-branches (Figure 2A). *Arabidopsis* PLP3 is required for tubulin folding during microtubule assembly and its impairment results in disrupted microtubule arrays, defective cytokinesis and disoriented cell growth [34,35].

Initial experimentation showed that the *Sl*TXND9/PepMV TGBp1 complex can be detected in the cytoplasm of agroinfiltrated *N. benthamiana* epidermal cells and, as for the CAT1/PepMV TGBp1 protein complex [18], the transcriptome and fractionated protein samples from PepMV-infected leaves did not contain elevated levels of *Nb*TXND9 mRNA or protein, when compared to those of healthy plants. However, *Nb*TXND9 appears to accumulate in the P1 and P30 pellets suggesting an association with membranes and organelles. Immuno-gold labelling electron microscopy also indicated the presence of *Nb*TXND9 close to plasmodesmata and the endoplasmic reticulum. In the case of PVX, the TGBp2-interacting TIP host factor that links PVX movement with callose degradation is also localized in the cytoplasm of infected cells [10]. The current potexvirus theory whereby the CP-vRNA-TGBp1 complex moves from cell-to-cell [36] may include a specific TXND9:TGBp1 interaction to physically assist movement of the complex towards or/and through plasmodesmata. This hypothesis is in accordance with the phylogenetic relatedness of TXND9 to PLP3, which functions in microtubule assembly [35] and the well-known role of microtubules in plant virus movement [37]. 

Recently, it has been shown that TRXs are able to move from cell-to-cell through plasmodesmata and play a role in intercellular communication [38,39]. Interestingly, the *Nb*TRXh2 homologue located in the plasma membrane has been reported to bind BaMV TGBp2 and hinder virus movement in *N. benthamiana* plants [9]. A hypothesis whereby two members of the TRX superfamily (in)directly interact so that *Nb*TXND9 overcomes the hindrance of potexvirus movement caused by *Nb*TRXh2, merits further investigation.

In a previous study [18], an increased efficiency in ROS scavenging by CAT1 as a result of its interaction with TGBp1 was interpreted as a PepMV strategy to avoid increased ROS levels and consequently to suppress defense-related host gene expression. Our present data do not allow conclusions to be drawn as to whether both *Nb*CAT1 and *Nb*TXND9 constitute elements of the same oxidative stress signaling pathway in PepMV pathogenesis with TGBp1 being a common factor. Bearing in mind that the complete TRX redox hallmark motif is not present in TXND9, biochemical studies focusing on its ability to scavenge ROS and an examination of its possible role in potexvirus movement would be meaningful.

## 4. Materials and Methods 

### 4.1. Plant Materials, Growth Conditions and Plant Inoculations

*S. lycopersicum* cv. Boludo tomato (Seminis Vegetable Seeds Europe, Enkhuizen, The Netherlands) and *N. benthamiana* tobacco plants were used in this investigation. *N. benthamiana* seeds were sown on Murashige-Skoog medium and germinated for 10 days at 25 °C under a 16:8 h photoperiod. Subsequently all seedlings were grown under standard conditions in growth chambers. The Spanish PepMV-Sp13 mild isolate (EU type), was used to mechanically inoculate tomato and *N. benthamiana* plants as previously described [16,17,18]. CMV was also used for *N. benthamiana* inoculations as a non-potexvirus.

### 4.2. Tomato cDNA Library Screening in Yeast

To screen for tomato proteins that interacted with PepMV TGBp1, a tomato cDNA library cloned into the pJG4-5 vector [40] was used, resulting in expression of the cDNAs as fusions with a portable transcriptional activation domain (the acid blob B42AD) and a hemagglutinin (HA) epitope under the control of the GAL1 promoter [41]. The “bait” construct—PepMV TGBp1 cloned to pGILDA (Clontech Laboratories Inc., Palo Alto, CA, USA)—has been previously described [18] and results in expression of a p26-LexA DNA-binding domain fusion, also under the control of the GAL1 promoter. *Saccharomyces cerevisiae* strain EGY48 cells were co-transformed with pGILDA-p26 and the pJG4-5/tomato cDNA library and plated onto Glucose/Complete Medium lacking histidine and tryptophan (Glu/CM-H,W) and plated onto Galactose-Raffinose/CM lacking histidine, tryptophan and leucine (Gal-Raff/CM-H,W,L) as previously described [16,18]. Co-transformations of either pGILDA-p26 with pJG4-5, or pGILDA with pJG4-5/cDNA library, were used as negative controls. Putative positive library plasmids were isolated, reintroduced back into the original reporter strain and co-transformed with EGY48 yeast cells in the presence or absence of LexA-TGBp1 onto selective medium, in order to confirm the interactions. The inserts of the plasmids that gave positive reactions were analyzed by sequencing.

### 4.3. Sequence and Phylogenetic Analysis

For sequence analysis the CLUSTAL X alignment software was used [42]. The phylogenetic analysis was inferred using the Neighbor-Joining method [43]. The bootstrap consensus tree inferred from 1500 replicates is taken to represent the evolutionary history of the taxa analyzed [44]. Branches corresponding to partitions reproduced in less than 50% of the bootstrap replicates are collapsed. The percentage of replicate trees in which the associated TRX domains clustered together in the bootstrap test (1500 replicates) are shown next to the branches. The trees are drawn to scale, with branch lengths in the same units as those of the evolutionary distances used to infer the phylogenetic trees. The evolutionary distances were computed using the Poisson correction method [45] and are in the units of the number of amino acid substitutions per site. All positions in all trees, with less than 0% site coverage, were eliminated. That is, fewer than 100% alignment gaps, missing data, and ambiguous bases were allowed at any position. Evolutionary analyses were conducted in MEGA5 [46].

### 4.4. In Vitro Protein Binding—Pull Down Assay

To confirm in vitro the PepMV TGBp1-*Sl*TXND9 interaction, bacterially expressed proteins of *Sl*TXND9 and TGBp1 were used as described before [18]. The *Sl*TXND9 cDNA was obtained by reverse transcription from the RNA isolated from tomato cv Boludo leaves. A total of 1 μg of total RNA was treated with DNase I (Thermo Scientific, Waltham, MA, USA), and then reverse-transcribed using PrimeScript™ Reverse Transcriptase (Takara Bio Inc., Nojihigashi, Japan) using an dT_18_ oligonucleotide primer according to the manufacturers’ instructions. The cDNA as amplified using the primers *Sl*TXND9-G-F and *Sl*TXND9-G-R and cloned into the plasmid vector pGEMTeasy (Promega, Madison, WI, USA) as directed to create pG-*Sl*TXND9. The 639 nt insert was sequenced to verify its identity with the partial gene sequences identified from the yeast 2-hybrid screen. The *Sl*TXND9 cDNA was PCR-amplified using primers *Sl*TXND9-EXP-F and *Sl*TXND9-EXP-R and cloned into the expression vector pRSF1b (Novagen, Madison, WI, USA) vector via *Bam*HI*-Xho*I sites, to generate the plasmid p*Sl*TXND9-S:tag, and with the primers *Sl*TXND9-MAL-F and *Sl*TXND9-MAL-R with cloning into pMALc2x (New England BioLabs, Ipswich, MA, USA) via the *Bam*HI-*Pst*I sites, to generate pMBP-*Sl*TXND9. The TGBp1 ORF had been cloned into pMALc2x previously [18]. The His-*Sl*TXND9-S:tag and maltose-binding protein MBP-TGBp1 fusions were expressed in *E. coli* BL21-(DE3) cells and following sonication the fusion proteins were purified from the soluble fractions using S:tag or amylose resin according to the manufacturers’ instructions. The in vitro binding assay was carried out as before [18]. The primers used are listed in Supplementary Data (Table S1).

### 4.5. BiFC Assay

The pCYFP-*Sl*TXND9 and pNYFP-TGBp1 constructs were transformed into *Agrobacterium* and co-infiltrated into fully expanded leaves of *N. benthamiana* together with the *Tomato bushy stunt virus* (TBSV) p19 RNA-silencing suppressor, to study in planta interactions, as described before [16]. The plant binary vectors used in BiFC experiments were pSPYNE-35S and pSPYCE-35S, that allow the expression of the proteins of interest as fusions to the N-(NYFP) or C-terminal half (CYFP) of YFP together with a c-myc (pSPYNE-35S) or HA (pSPYCE-35S) affinity tag [26]. The pNYFP-TGBp1 has been described previously [18] while generation of the pCYFP-*Sl*TXND9 construct was achieved by PCR amplification of *Sl*TXND9 from the cloned cDNA using the primers *Sl*TXND9-YFP-F and *Sl*TXND9-YFP-R (Table S1), and cloned into the binary vector pSPYCE-35S via the *Asc*I-*Xho*I sites. The bZIP63 transcription factor was used as a positive control and combinations of pSPYNE-35S (pNYFP) and pSPYCE-35S (pCYFP) plasmids with plant- and viral-constructs, respectively, were used as negative controls. Images were recorded on SP2 and SP5 (Leica Microsystems, Heidelberg, Germany) confocal fluorescence laser scanning microscopes, and the YFP fluorescence was visualized with excitation at 514 nm and emission at 535–545 nm from the epidermal cell layers at 3 to 4 dpi.

### 4.6. Plant Response to PepMV Inoculation

To evaluate any alterations at the mRNA transcription level, identical volumes of PepMV inocula were used to inoculate *N. benthamiana* leaves, while control plants were mock-inoculated with phosphate buffer (0.5 M, pH 7.0). At 2, 4, 6, 8, and 10 dpi, total RNA was extracted from PepMV- and mock-inoculated local and systemic leaves and gene expression was determined using qRT-PCR in a StepOne^TM^ Real-Time PCR system (Applied Biosystems, Foster City, CA, USA). cDNA synthesis was carried out, as described above, for each time point and used as the templates for real-time PCR in 10 μL reactions using a master mix consisting of gene specific primers and Kapa SYBR^®^ Fast qPCR Master Mix (Kapa Biosystems, Wilmington, MA, USA). Following denaturation at 95 °C for 10 min, 40 cycles of denaturation at 95 °C for 15 s, annealing at 60 °C for 20 s and extension at 72 °C for 20 s, were applied. A melting curve analysis protocol was executed in the temperature range from 60 to 95 °C. The specific primers *NbSl*TXND9-qF and *NbSl*TXND9-qR (Table S1) were designed using the PRIMER3 software (Whitehead Institute for Biomedical Research, www-genome.wi.mit.edu/cgi-bin/primer/primer3.cgi/), and EF1a was used as the reference gene [47]. Data were analyzed using the 2^−ΔΔ*C*T^ method [48] and presented as relative levels of gene expression.

### 4.7. Antiserum Production and Processing

Cultures of *E. coli* BL21/DE3 transformed with either pRSF-*Sl*TXND9 or pMAL-*Sl*TXND9 were induced with 1 mM IPTG and grown on for 3 h before cell collection via centrifugation and sonication in 10–20 mL column buffer (CB: 25 mM Tris-HCl, 50 mM NaCl, 1 mM EDTA; pH 7.8). Overexpressed His-*Sl*TXND9-S and MBP-*Sl*TXND9 that accumulated in the soluble fractions were purified using affinity column chromatography (His-Trap column, GE Life Sciences) and amylose resin (New England Biolabs, Ipswich, MA, USA), respectively. Both proteins were subsequently purified via ion exchange chromatography (Q column, GE Life Sciences, Wien, Austria) according to the manufacturer’s instructions, desalted by dialysis against 0.5 × CB and lyophilised. A total of 1 mg of highly pure His-TRX-S protein was used to immunize a rabbit in order to produce a polyclonal antiserum, from which specific IgG was purified using a multi-stage purification process: Total IgG was selected from the serum using a protein-A column, anti-his IgG was depleted using a his-tagged column, and *Sl*TXND9 IgG was selected on the basis of its cross-reactivity with MBP-*Sl*TXND9 (David’s Biotechnologie, Regensburg, Germany).

### 4.8. Subcellular Fractionation

Proteins from healthy and PepMV infected *N. benthamiana* leaves were fractionated as described before [49]. The fractions examined were cell wall (CW), 1000× *g* pellet (P1), 30,000× *g* pellet (P30) and 30,000× *g* centrifugation supernatant (30S). Each fraction was for boiled 5 min in 1 volume of appropriate sample buffer, before resolution on a 12% SDS-PAGE gel, Western blotting and development with an antiserum raised against PepMV CP, host interactor proteins Hsc70 and CAT and the purified α-*Sl*TXND9 IgG. 

### 4.9. Immuno-Gold Labelling and Electron Microscopy 

Leaves from healthy, inoculated, and systemically-infected PepMV *N. benthamiana* plants were collected 4 dpi, together with those from CMV-infected control plants. Small pieces of leaf (0.1 × 1 cm^2^) were processed for immuno-gold labelling (IGL). Fixation was with 1% glutaraldehyde + 1% para-formaldehyde (*v*/*v*) in 0.1 M sodium phosphate buffer (pH 7.2) for 16–24 h at 4 °C, with washing three times (10 min) in the same buffer. Subsequently the samples were dehydrated in an alcohol series (30% to 100%) before being embedded in Lowicryl K4M resin (Polysciences, Inc., Warrington, PA, USA) in a cold chamber (−20 °C) with polymerization induced by ultraviolet light, according to suppliers’ protocols. Ultra-thin sections (70 nm) were produced using a Reichert-Jung Ultracut E microtome (Leica Microsystems, Mannheim, Germany) and mounted on Formvar^©^ carbon-coated gold grids (200 mesh). IGL analysis using the α-*Sl*TXND9 IgG produced in this study was according to the protocol of Medina et al. [50]. The secondary antibodies were goat-anti-rabbit IgG conjugated with 15-nm gold (Electron Microscopy Sciences, Hatfield, PA, USA). 

## Supplementary Materials

Supplementary materials can be found at http://www.mdpi.com/1422-0067/19/12/3747/s1.
