# Supplementary material for: A Thioredoxin Domain-Containing Protein Interacts with Pepino mosaic virus Triple Gene Block Protein 1
Source: Int J Mol Sci. 2018 Nov 25;19(12):3747. doi: 10.3390/ijms19123747 (PMC6320799; doi:10.3390/ijms19123747)
Supplement: Supplementary file 1 [file ijms-19-03747-s001.pdf]

A Thioredoxin Domain-Containing Protein Interacts with *Pepino mosaic virus* Triple Gene Block Protein 1

Matthaios M. Mathioudakis, Souheyla Khechmar, Carolyn A. Owen, Vicente Medina, Karima Ben Mansour, Weronika Tomaszewska, Theodore Spanos, Panagiotis F. Sarris and Ioannis C. Livieratos

Table S1. Primer pairs used for cloning and sub-cloning of *Sl*TXND9 and for real-time PCR quantitation of *Nb*TXND9 mRNA expression

Primer pair name	-F	-R
*Sl*TXND9-G	ATGGAGAATGCGGTTCAAGAGA	CTCCGAGTCGCACGAGTCG
*Sl*TXND9-EXP	**GGATCCC**ATGGAGAATGCGGTTCAAGAGA	**CTCGAG**CTCCGAGTCGCACGAGTCG
*Sl*TXND9-YFP	**GGATCC**ATGGAGAATGCGGTTCAAGAGA	**GGCGCGCC**CTCCGAGTCGCACGAGTCG
*Sl*TXND9-MAL	**GGATCC**ATGGAGAATGCGGTTCAAGAGA	**CTGCAG**CTCCGAGTCGCACGAGTCG
*NbSl*TXND9-q	TTGTTAAGGCTAGTGACCGTGTT	CTATATGCTGTTTTGCCAGTATGC

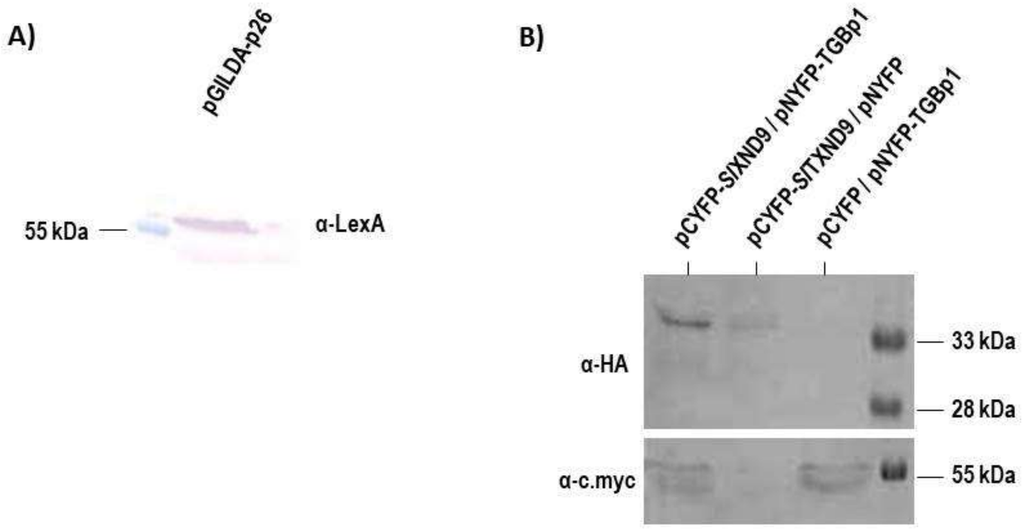

Figure S1. Immunoblot analysis of the fusion proteins *LexA*-TGBp1, CYFP-SlTXND9 and NYFP-TGBp1. **(A)** Immunoblot analysis to confirm the expression of the *LexA*-TGBp1 fusion protein (the expression product of plasmid pGILDA-p26) in yeast. This construct was used as the bait to screen the tomato cDNA library subcloned into the pJG4-5 vector. Western blot of SDS-PAGE resolved total proteins from pGILDA-p26-transformed *Saccharomyces cerevisiae* EGY48 cells probed with α-LexA antiserum revealed a fusion protein of the expected size (58 kDa). **(B)** Immunoblot analysis to detect the *Agrobacterium*-mediated transient expression of CYFP-*Sl*TXND9 and NYFP-TGBp1 in *Nicotiniana benthamiana* plants for the bimolecular fluorescent complementation assay (BiFC). Total protein extracts from *N. benthamiana* leaves co-infiltrated with pSPYNE-35S-TGBp1 (encoding NYFP-TGBp1, 55kDa) and pSPYCE-35S-*Sl*TXND9 (encoding CYFP-*Sl*TXND9, 35 kDa), or each fusion separately in conjunction with the empty vector encoding the complementary YFP fragment, harvested at 3 days post-inoculation (dpi), were used to produce western blots that were probed with either α-c-myc (pSPYNE) or α-HA (pSPYCE) that recognize the affinity tags on the fusion proteins, to confirm their expression.

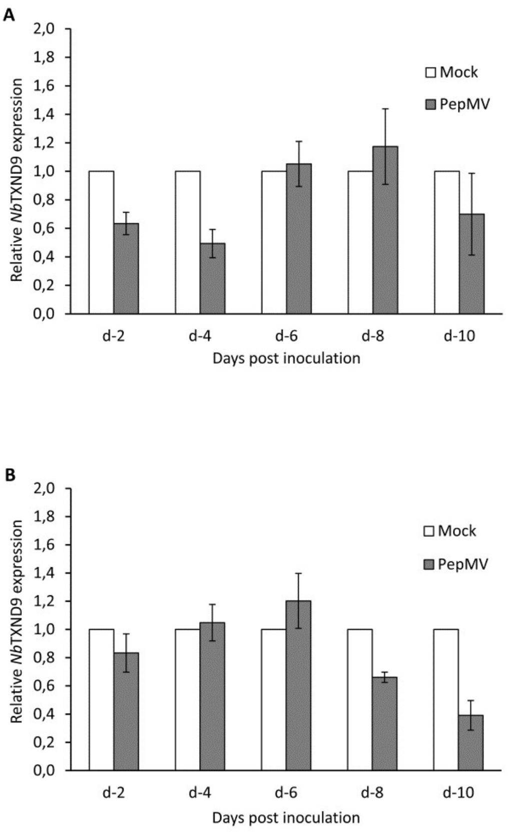

Figure S2. Accumulation of SlTXND9 mRNA in PepMV- and mock-inoculated *Nicotiniana benthamiana* plants. *N. benthamiana* plants were inoculated with PepMV or mock-inoculated, and the *Nb*TXND9 mRNA expression levels were evaluated by quantitative RT-PCR in locally-inoculated **(A)** or systemically-infected apical **(B)** leaves at 2, 4, 6, 8, 10 dpi. The expression of the housekeeping gene Elongation Factor 1a was used as a normalization control.

The mean values from three independent experiments are presented with the mean value of the mock-inoculated expressed as 1. Error bars represent standard deviation (±) of the mean values.

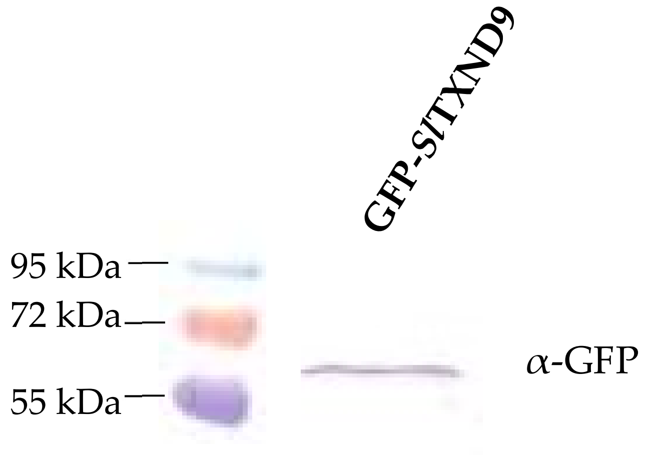

Figure S3. Immunoblot analysis to detect the agrobacterium-transiently expressed GFP-*Sl*TXND9 fusion protein in total protein extracts from *N. benthamiana* plants.
